# Malignancy in dermatomyositis: A retrospective paired case–control study of 202 patients from Central China

**DOI:** 10.1097/MD.0000000000021733

**Published:** 2020-08-21

**Authors:** Lili Chang, Lina Zhang, Haiquan Jia, Zhiyong Nie, Lei Zhang

**Affiliations:** a Department of Oncology, the Fourth Affiliated Hospital of Henan University of Science and Technology, Anyang Cancer Hospital, Anyang, Henan; b Department of Breast Surgery, Tianjin Medical University Cancer Institute and Hospital, Tianjin; c Department of General Surgery, the Fourth Affiliated Hospital of Henan University of Science and Technology, Anyang Cancer Hospital, Anyang; d Department of Rheumatology, the First Affiliated Hospital of Zhengzhou University, Zhengzhou, Henan, China.

**Keywords:** albumin, dermatomyositis, interstitial lung disease, malignancy, predictive factors

## Abstract

Dermatomyositis (DM) is an idiopathic inflammatory myopathy that is closely related to malignant diseases. Our study aims to investigate the incidence and predictive factors for occurrence of malignancy among DM patients from Central China.

We performed a retrospective, paired, case–control study of 736 DM patients admitted to the First Affiliated Hospital of Zhengzhou University between 2010 and 2017. We paired the 65 patients with malignancy with age-matched and sex-matched patients without malignancy in a ratio of 1:2. Two hundred two patients were finally enrolled and their clinical and laboratory data were collected.

The incidence of malignancy in DM patients was 8.83% (65/736). Most malignancies were detected in the most recent 1 year before (9/65, 13.85%) or within 3 years after (40/65, 61.54%) the onset of DM. Males (35/65, 53.85%) and patients aged between 50 and 69 years (43/65, 66.15%) were prone to develop malignancies. Lung cancer (n = 11, 31.43%) was the most common malignancy in male patients, while for females, thyroid, breast and cervical cancer (n = 4 each, 13.33%) were more prevalent. Adenocarcinoma and squamous cell carcinoma (both 18/65, 27.69%) were the top two most common pathological types. Univariate analysis demonstrated that Gottron's sign (*P* = .02), dysphagia (*P* = .04), albumin (ALB) reduction (*P* = .003), aspartate aminotransferase (AST, *P* = .03), creatine kinase-MB (*P* = .02), absence of fever (*P* = .02), arthralgia (*P* = .04) and interstitial lung disease (ILD, *P* = .05) were closely related to the occurrence of malignancy. Multivariate analysis revealed the independent risk factors of ALB reduction (odds ratio = 1.546, *P* = .04) and the protective factor of ILD (odds ratio = 0.349, *P* = .003). There was no significant difference in the follow-up period between patients with and without ILD (*P* = .38).

ALB reduction and the absence of ILD were the risk factors for malignancy in DM patients. The protective mechanism of ILD for DM patients needs further study.

## 1 Introduction

Dermatomyositis (DM) is a rare systemic autoimmune disease that is characterized by skin rash and worsening proximal muscle weakness. It is an idiopathic inflammatory myopathy, which includes three main subtypes: DM, polymyositis and inclusion-body myositis. In 1916, Stertz^[1]^ first reported inflammatory myopathy in a patient with gastric adenocarcinoma. Since then, numerous studies have shown the high cancer incidence among patients with idiopathic inflammatory myopathy, especially DM.^[1–8]^

DM with coexistent malignancy ranges from 6% to 60% of all the DM cases.^[9–12]^ Five-year survival of adult DM patients is 60% to 90%,^[13]^ and malignancy is the primary cause of death. It is likely that prognosis in patients with coexistent malignancy is poor due to the cancer, therefore, it is necessary to analyze the relationship between them.

The onset of DM and malignancy is closely linked. The pathogenetic mechanism is believed to be as follows: immunosuppressive therapy in DM disease might induce the incidence of malignant disease^[7]^; the immunological response to internal malignancy might prompt the onset of DM^[14,15]^; and heightened surveillance also increases the malignancy detection rate. Recently, DM has been considered to be a paraneoplastic syndrome, and a clinical manifestation of the remote effects produced by tumor metabolites or other products.^[16]^ DM improves after treatment of cancer, otherwise muscle and/or skin changes would occur at relapse of malignant disease, further supporting that it is a paraneoplastic phenomenon.^[17]^

Previously identified risk factors for malignancy include older age, male gender, elevated erythrocyte sedimentation rate, reduced albumin (ALB) level, presence of dysphagia, cutaneous vasculitis, Gottron's sign, heliotrope rash, and rapid-onset myositis.^[5,9,12]^ Unlike most research designs, in which the total DM cohort was the final studied patients, we made the malignant patients as basis for randomly selecting nonmalignant patients according to age and gender in a ratio of 1:2. Clinical and laboratory characteristics of those patients were analyzed to assess the underlying predictive factors for occurrence of malignancy, which might help with early cancer detection and potentially reduce mortality for DM patients.

## 2 Methods

### 2.1 Patients

**Figure 1:**
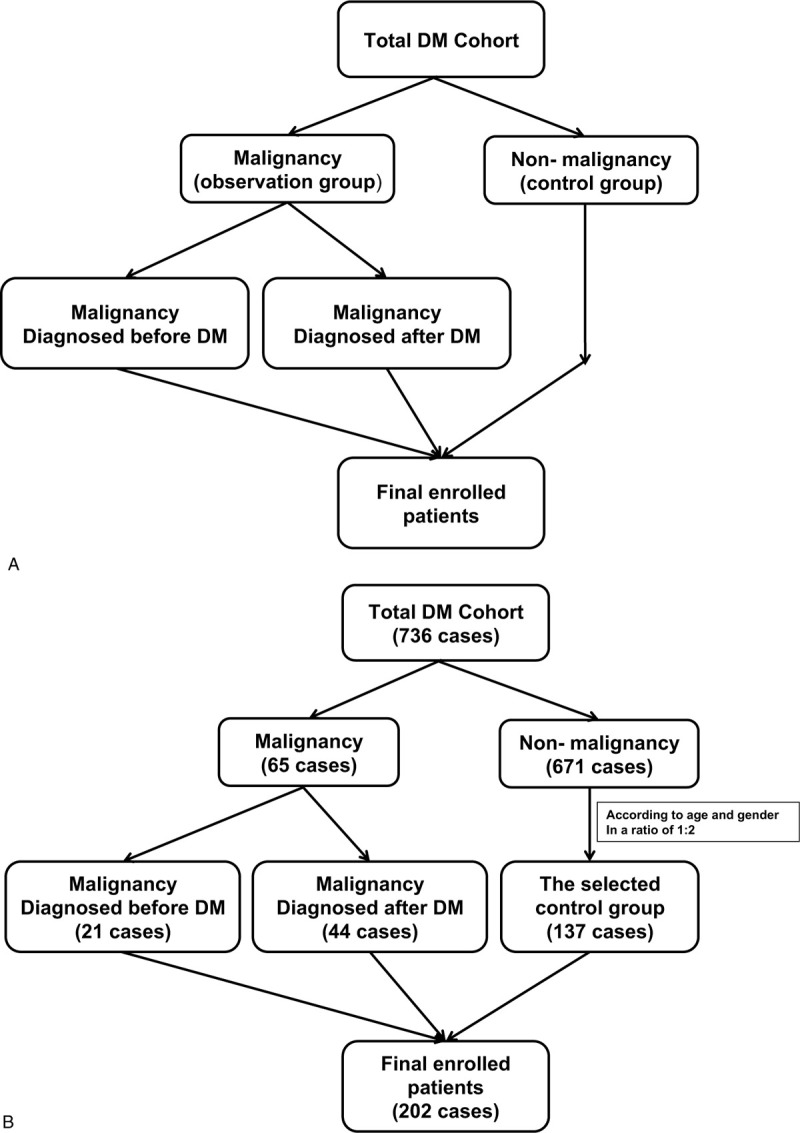
Flowchart for patients screening. A: Total dermatomyositis cohort was the final studied patients, which were divided into observation (malignant) and control (nonmalignant) groups. B: Malignant patients were the observation group, who acted as the basis for randomly selecting nonmalignant patients according to age and gender in a ratio of 1:2.

We retrieved medical records of 736 DM patients admitted to the First Affiliated Hospital of Zhengzhou University between January 2010 and December 2017. Unlike the traditional classification of patients into malignant and nonmalignant groups, we made malignant patients as the observation group and select nonmalignant patients according to age and gender as the control group. We used the systematic sampling method and matched the 65 patients with malignant disease with another 137 DM patients in nonmalignant group (Fig. 1A and B). Two hundred two patients were finally enrolled, among which 7 patients overlapped with Sjogren syndrome, 2 patients overlapped with rheumatoid arthritis, 1 patient combined with vasculitis and another 1 patient combined with vitiligo. Only one DM patient with Sjogren syndrome and another DM patient with rheumatoid arthritis finally developed into malignant diseases. Diagnosis of DM was based on the criteria of Bohan and Peter.^[18]^ Cancer was confirmed by corresponding pathology. Patients younger than 18 years of age were excluded as the criteria for them are different.^[19]^ Our study was approved by the Institutional Review Board of the First Affiliated Hospital of Zhengzhou University (Henan, China, approval number: 2019-KY-241). Written informed consent was not obtained from each patient due to the retrospective nature of the study. All patients in our study were followed up from the onset of DM until loss of follow-up or death or otherwise until 23 January 2019.

### 2.2 Data collection

We collected clinical and laboratory data on the onset of DM, gathered from admission and outpatient clinic records. Parameters assessed included general data (age, gender, smoking, drinking, hypertension, diabetes, skin lesions, and genetic history), clinical characteristics [fever, Gottron sign, heliotrope rash, neck V–shaped rash, myalgia, proximal muscle weakness, arthralgia, mechanic hand, dysphagia, Raynaud phenomenon, and interstitial lung disease (ILD)], laboratory data (blood routine examination, hepatic and renal function tests, inflammatory indices, muscle enzymes in serum, connective tissue disease associated autoantigens, myositis–specific autoantibodies, and tumor markers), diagnostic time of DM and cancers, type and pathological classification of tumor, time interval between the onset of DM and malignancies, follow-up results, and time and causes of death. The survival of patients was calculated from the date of DM diagnosis to the date of last follow-up or death.

### 2.3 Statistical analysis

Two-tailed Student *t* test or Mann–Whitney *U* test was adopted to compare the normally distributed continuous variables. Categorical variables were compared by χ^2^ test or Fisher exact test. Variables with *P* < .05 in univariate analysis were included in the multivariate models. The odds ratios (ORs) and 95% confidence interval were calculated by multivariate Cox proportional hazards regression analysis, through which independent risk factors were identified. All statistical calculations were carried out using SPSS version 20.0 (Chicago, IL). *P* < .05 was considered a statistically significant difference.

## 3 Results

### 3.1 Time-interval

**Figure 2:**
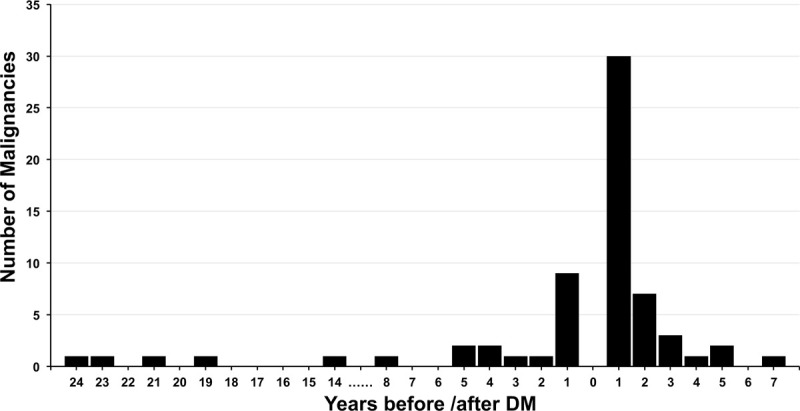
Temporal association between onset of dermatomyositis and malignancy.

Forty-four (44/65, 67.69%) malignancies were detected after the onset of DM: 30 (46.15%) were diagnosed within 1 year and 40 (61.54%) within 3 years. Twenty-one (21/65, 32.31%) cancers were diagnosed before the occurrence of DM: 9 (13.85%) were within the most recent 1 year, 11 (16.92%) were within 3 years and 15 (23.08%) within 5 years. Time-intervals between the onset of DM and malignancy are illustrated in Figure 2.

### 3.2 Factors associated with malignancy

Univariate analysis of risk factors for malignancy in DM patients demonstrated that Gottron's sign (35.77% vs 52.31%; *P* = .02), dysphagia (26.28% vs 38.46%; *P* = .04), ALB reduction (43.38% vs 47.69%; *P* = .003), AST (51.82% vs 58.46%; *P* = .03), creatine kinase–MB (48.91% vs 58.46%; *P* = .02), absence of fever (35.04% vs 20.0%; *P* = .02), arthralgia (33.58% vs 16.92%; *P* = .04) and ILD (42.34% vs 23.08%; *P* = .05) were the predictors of malignancy. We entered the significant factors above into the Cox regression for multivariate analysis. ALB reduction (*P* = .04) was an independent risk factor, while the presence of ILD (*P* = .003) was the only protective factor (Tables 1–3).

**Table 1:**
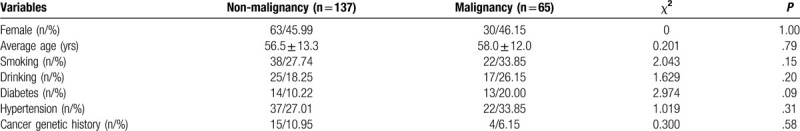
Clinical features of 65 malignancy-associated dermatomyositis patients and 137 matched controls.

**Table 2:**
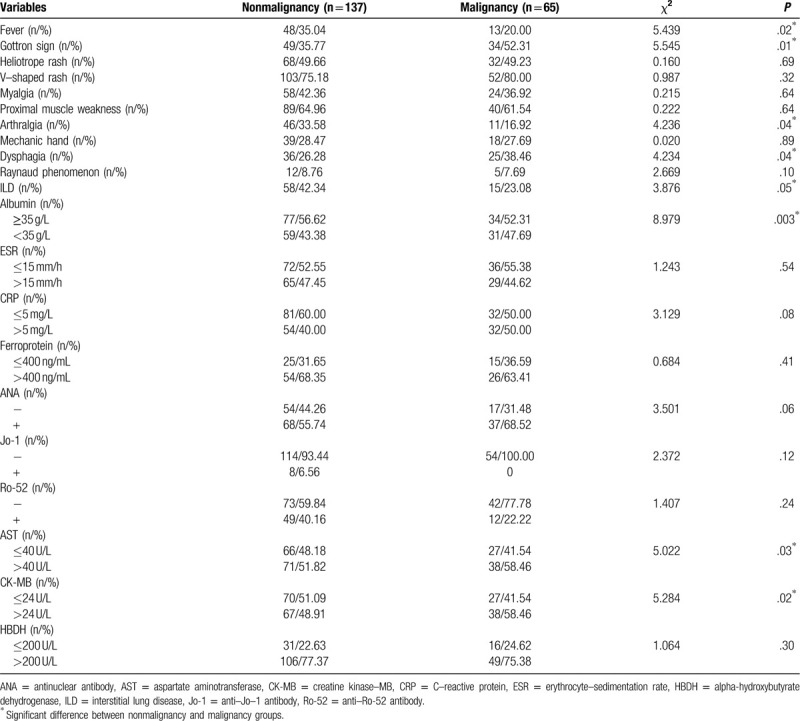
Univariate analysis of factors potentially associated with malignancy in 202 patients.

**Table 3:**
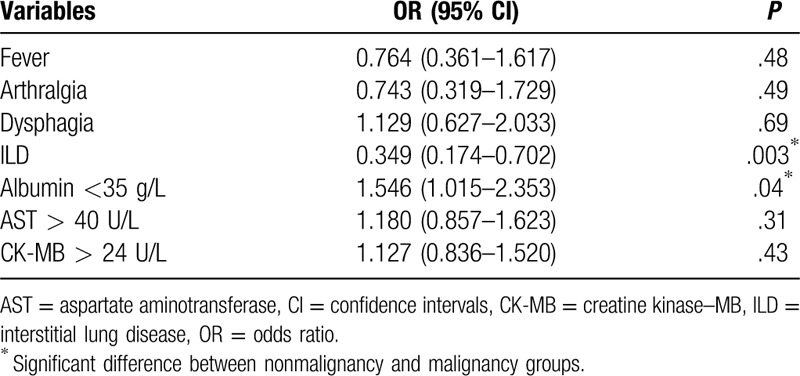
Multivariate analysis of factors associated with malignancy in 202 patients.

As our research was a paired case–control study, in which age and gender were the pairing factors, the age and gender composition of the malignant and nonmalignant groups were the same. We only analyzed the data for 65 malignant patients as a representation. As a result, the cancer incidence increased with age but decreased beyond 70 years. The peak point was located between 50 and 69 years (43/65, 66.15%) (Table 4).

**Table 4:**
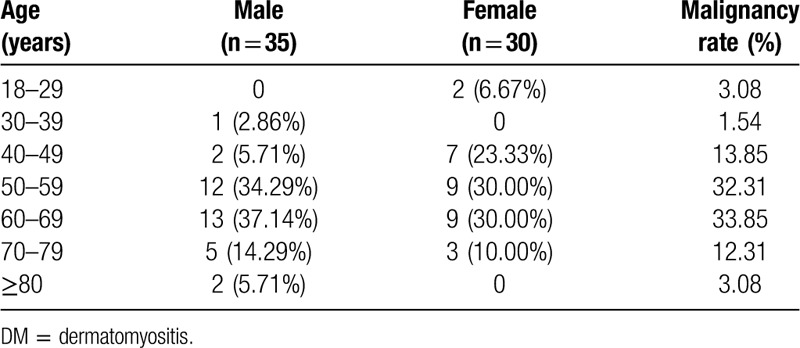
Malignancy rates in different age groups among 65 DM patients.

### 3.3 Tumor species and pathological types

Types of malignant diseases among the 65 DM patients were as follows: 13 lung cancer, 7 esophageal carcinoma, 5 thyroid cancer, 4 breast cancer, 4 nasopharyngeal carcinoma, 4 gastric cancer, 4 cervical cancer, 3 hepatocellular carcinoma, 2 malignant melanoma, 2 rectal cancer, 2 uterine carcinoma, 2 oral cancer, 2 bladder cancer, 2 malignant hematological disease, 1 laryngeal carcinoma, 1 malignant thymoma, 1 colorectal cancer, 1 small intestinal carcinoma, and another 4 unidentified rib or lymph node metastases. Lung cancer had the highest incidence (13/65, 20.0%), followed by esophageal carcinoma (n = 7, 10.77%) and thyroid cancer (n = 5, 7.69%). The top 2 most common malignant diseases were lung cancer (n = 11, 31.43%) and esophageal carcinoma (n = 5, 14.29%) in men while thyroid, breast and cervical cancers (n = 4 each, 13.33%) were more common in women. Adenocarcinoma and squamous cell carcinoma (both n = 18, 27.69%) were the predominant pathological types; for men it was squamous cell carcinoma (n = 11, 31.43%) and for women it was adenocarcinoma (n = 10, 33.33%) (Tables 5 and 6).

**Table 5:**
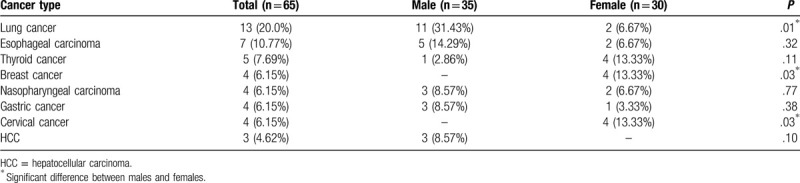
Top 8 types of cancer among 65 malignant patients.

**Table 6:**
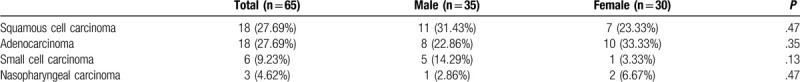
Top 4 pathological types of cancer among 65 malignant patients.

### 3.4 Treatment

We collected the information on the medication use of patients in our study. Data of detailed medication use process for patients lost to follow-up or had been dead could not be collected precisely. Through the analysis of drug compositions in our study, we found that glucocorticoids were the mainstay of initial management; for the 44 malignant cases with DM first onset, the number of patients treated with glucocorticoid only was 20 (20/44, 45.5%), the number of glucocorticoid combined with chloroquine was 9 (9/44, 20.5%), and the number of glucocorticoid combined with tripterygium wilfordii was 3 (3/44, 6.8%); while for the 137 nonmalignant cases, the number of patients treated with glucocorticoid only was 63 (63/137, 46.0%), the number of glucocorticoid combined with chloroquine was 22 (22/137, 16.1%), and the number of glucocorticoid combined with cyclophosphamide was 14 (14/137, 10.2%).

## 4 Discussion

DM is a type of idiopathic inflammatory myopathy. The incidence of adult DM is 5.0 to 8.9 per million persons.^[20,21]^ The prevalence of malignancy in DM patients in western countries ranges from 8.6% to 32.0%,^[11,14]^ while in Asians it is 3.8% to 56.0%.^[9,22]^ In our study, the rate was 8.83%, which was in accordance with previous reports. Five-year survival of DM patients ranges from 60% to 90%, while it is only 10% to 56% for patients with malignant diseases.^[13,23,24]^

Malignancies might be found around the time of onset of DM. A recent study from Northern China reported that 69.77% of malignancies were detected before or after DM.^[25]^ A report from Hungary^[26]^ implied that 64.8% of the malignant cases appeared within 1 year after DM diagnosis. A large meta–analysis from Canada found that 17.29% of DM patients had malignancies within 1 year and 2.7% within 2 to 5 years of DM diagnosis.^[27]^ Of the 65 patients with malignant disease in our study, 30 (46.15%) cases were detected within 1 year after diagnosis of DM, and the 3-year diagnosis rate approached to 61.54% (40/65). Cancer occurring before myositis accounted for 32.31% (21/65), and 9 (42.86%) of these 21 cases were discovered within 1 year before DM. This reminded our clinicians that for newly diagnosed DM patients, we should first clarify whether it is complicated with malignant diseases, and previous symptoms and signs and physical examination results within the last year would be useful. Besides, DM patients should undergo lifelong regular physical examination, and especially within the first 3 years after the onset of DM.

Previously reported malignant risk factors include older age, male gender, dysphagia, cutaneous necrosis, rapid-onset myositis, cutaneous vasculitis, Gottron sign, heliotrope rash, reduced serum ALB, and elevated ALT, AST, lactate dehydrogenase (LDH), erythrocyte sedimentation rate, and C-reactive protein, while protective factors include ILD, arthralgia, Raynaud and positive anti–Jo–1.^[5,9,12,28]^ To our knowledge, few of those studies were paired case-controlled. Unlike the most classification of the total DM cohort into malignant and nonmalignant groups, we compared the malignant patients with age-matched and sex-matched control patients who were selected from the pool of 671 nonmalignant patients in a ratio of 1:2. By doing this, the interference from age and gender was minimized and an impartial assessment of the possible risk factors was obtained. We confirmed the risk factors of low ALB and the protective factor of ILD. Although it had been assumed that cancer patients were prone to severe muscle weakness, leading to swallowing muscle involvement,^[29]^ we did not find that dysphagia was independently associated with malignancy. More studies are needed to further explore the potential mechanisms.

ALB is an inflammatory marker. An important finding of our study was the association between low ALB and the risk of malignancy. As reported,^[30]^ hypoalbuminemia may reflect both poor inflammatory reaction and patients’ poor general status. DM patients with prominent inflammatory marker ALB and well-preserved muscle strength would be more likely to get a subsequent ILD complication,^[31]^ while ILD was suggested a negative correlation to malignancy in DM. There may be additional mechanisms by which reduced ALB increases the malignancy risk in myositis. Further studies are needed to clarify the mechanisms. In our study, genital system tumor and breast cancer were reported as the 2 main types of cancers associated with DM for women, and lung cancer was the most popular for men. These pathological types may have some influence on the outcomes.

ILD is a common complication of DM. An acute type of ILD could leads to increased morbidity and mortality because of resistance to conventional therapy. As reported, the incidence of ILD among DM patients ranged from 11% to 74%.^[9,32]^ In our study, 36.14% developed ILD. The OR of 0.349 indicated that the presence of ILD might diminish the risk of coexistent malignancy by 65.1%. Recent analyses have shown that ILD is associated with a relatively lower risk of malignancy in patients with myositis.^[33]^ One possible explanation is related to the presence of autoantibodies against aminoacyl-transfer synthetases, which is a common feature of ILD and appears to be protective against malignancies.^[30]^ No patient with malignancies with autoantibodies against aminoacyl-transfer synthetases was found in our study. Another explanation is that ILD has a poor prognosis, and patients might die before the detection of malignancy. However, in our study, patients with and without ILD had similar follow-up periods (29.00 ± 28.07 months vs 32.00 ± 27.02 months; *P* = .38), which was consistent with a study from Korea.^[34]^ A recent meta-analysis from China even failed to show that ILD is protective against underlying malignancy.^[5]^ The protective mechanism of ILD for DM patients from cancer needs further study.

Age and gender have been considered to influence the development of malignancies among DM patients. A report from Scotland^[6]^ revealed that patients aged 45 to 75 years had an increased risk of cancer following the onset of DM. A study from Taiwan^[35]^ implied that risk of malignancy was evident in every age group, especially within patients aged 40 to 59 years and >80 years. Few studies have shown that men are more likely to develop cancer than women are,^[27,28]^ while other studies have reported the opposite conclusion.^[36,37]^ As patients in our study were matched according to age and gender, we only analyzed the age and gender composition of 65 patients in the malignant group. We found that patients aged 50 to 69 years accounted for a large proportion, reaching 66.15% (43/65), and men (35/65, 53.85%) were more common than women (30/65, 46.15%).

Patients without high levels of tumor markers could be found malignancies at the same month that DM was diagnosed. However, patients with an elevation of tumor markers could be detected malignancies 32 months after the onset of DM: one case of DM with a high level of carcinoembryonic antigen was found to have gastric cancer 16 months later; one case of DM with high levels of CA-211 and neuron–specific enolase was found to have nonsmall cell lung cancer 32 months after the DM diagnosis; another DM patient with a high level of CA-211 was detected to have NSCLC with a brain metastasis in only 2 months. Malignancies in our study were confirmed only by pathologies. Tumor markers, suspicious signs or symptoms suggestive of malignancies or computed tomography (CT) screening could imply the possibility of cancer but they could not lead to the diagnosis of cancer. Regular monitoring the fluctuation of tumor markers could have some clinical values for nonmalignant DM patients, especially for those undetected malignant diseases that had already metastasized.

Tumor location among DM patients varies according to geography and ethnicity. In Sweden, Finland and Denmark,^[38]^ cancers of the ovary, lung, pancreas and gastrointestinal tract and non–Hodgkin lymphoma are more dominant. In Scotland,^[6]^ cervical, ovarian and lung cancers were often involved. In Southern China, and other Asian countries such as Singapore, India and Taiwan, nasopharyngeal carcinoma, and cancers of lung, breast, gastrointestinal tract, ovary and liver are more common.^[39,40]^ In our current study, lung cancer came first, which was followed by cancers of the esophagus and thyroid. Unlike the high incidence in Southern China, nasopharyngeal carcinoma in the central plains ranked only fourth. There was also a sex difference. The predominant type of malignancy among men was lung cancer, whereas among women, it was thyroid, breast and cervix cancer. A study from the United States found that malignant diseases without suspicious signs or symptoms at the time of DM onset accounted for 6.8% of cases, and repeat CT scan was the most recommend method to reveal potential cancer.^[7]^ However, who merits such screening and the scanning frequency have not been clarified. Consistent with previous reports,^[41]^ adenocarcinoma and squamous cell carcinoma were more prevalent in our study.

One of the assumed pathogenetic mechanisms between DM and malignancy was that immunosuppressive therapy in DM disease might induce the incidence of malignant diseases. Until now there have not been any therapies approved for the treatment of DM by the European Medicines Agency based on randomized controlled trials.^[42]^ The core therapeutic approach remains oral corticosteroid administration, along with adjunctive steroid-sparing immunosuppressive agents, such as methotrexate, azathioprine, cyclosporine A, and mycophenolate mofetil. Multicenter and randomized trials, international collaborations and validated outcome measures are required to the development of optimal therapeutic alternatives in DM.

Our study had some of the inherent limitations of a retrospective clinical analysis. First, as patients were distributed in different departments, physicians might not have followed a standardized questionnaire to assess systematically and comprehensively the general condition of the DM patients. Results like myositis specific autoantibodies would even be lost, which might lead to information bias. Second, in our study, only cases with suspicious signs, such as symptoms suggestive of malignancies or higher tumor makers would be screened for cancer. The diagnostic tests were aimed at the corresponding organs or tissues. For solid carcinomas, the highest yield screening strategies were chest and pelvic CT scans, another less expensive modality was ultrasound. Methods to diagnose hematological malignancies were bone marrow biopsy and bone marrow aspiration. Positron emission computed tomography (PET-CT) scanning was seldom used due to the cost considerations. All those factors might lead to the decrease of tumor detection rate. In addition, patients in our study were recommended to take physical examinations annually. However, the role of repeat blind cancer screening among asymptomatic DM patients was not available. Third, the longest follow-up period from the onset of DM among patients with first-detected DM was 6.8 years, making it possible that some DM-associated malignancies had not yet been detected. Finally, these results represented only a small data set from our hospital, and selection bias might have existed.

In conclusion, the risk of developing cancer lasts for many years after the onset of DM. Investigation of malignancies in DM patients should be implemented lifelong, especially within the first 3 years. Newly diagnosed DM patients should be investigated for potential malignant diseases. Men and patients aged 50 to 69 years should undergo frequent cancer screening. For DM patients without suspicious signs or symptoms suggestive of malignancies, CT screening is so far the most effective method to discover malignancy in a short time; while for patients with clinical symptoms, diagnostic tests aimed at the corresponding organs or tissues are more appropriate. Sufficient attention should be paid to the decrease in serum ALB. Men should be vigilant about lung cancer, whereas women should be cautious about breast, thyroid and cervical cancer. The protective mechanism of ILD in DM patients needs further study. Future studies will be needed to identify the frequency and the role of blind cancer screening among DM patients, especially the ones without any symptoms. Long-term follow-up and more mechanistic studies are needed to promote the establishment of cancer screening guidelines.

## Author contributions

Lei Zhang and Lili Chang conceived and designed the research. Lili Chang analyzed the data and wrote the paper. Lina Zhang tributed writing assistance. Haiquan Jia and Zhiyong Nie collected and performed the registration of follow up data for all patients. All authors read and approved the final manuscript.
